# Association of Baseline Smoking Status with Long-Term Prognosis in Patients Who Underwent Percutaneous Coronary Intervention: Large Single-Center Data

**DOI:** 10.1155/2019/3503876

**Published:** 2019-05-05

**Authors:** Ru Liu, Zhan Gao, Huanhuan Wang, Xiaofang Tang, Lijian Gao, Ying Song, Jingjing Xu, Jue Chen, Shubin Qiao, Yuejin Yang, Runlin Gao, Bo Xu, Jinqing Yuan

**Affiliations:** Department of Cardiology, Fuwai Hospital, Chinese Academy of Medical Sciences and Peking Union Medical College, Beijing, China

## Abstract

**Objectives:**

This study analyzed a large sample to explain the association of baseline smoking state with long-term prognosis of coronary artery disease (CAD) patients who underwent percutaneous coronary intervention (PCI).

**Background:**

Data is limited up to now regarding whether smoker's paradox exists in Chinese population.

**Methods:**

A total of 10724 consecutive cases were enrolled from January to December 2013. 2-year clinical outcomes were evaluated among current smokers and nonsmokers. Major adverse coronary event (MACCE) included all-cause death, revascularization, myocardial infarction (MI), and stroke.

**Results:**

Current smokers and nonsmokers accounted for 57.1% and 42.9%, respectively. Current smokers were presented with predominant male sex, lower age, and less comorbidities. The rates of 2-year all-cause death were not significantly different among two groups. But the rate of stroke and bleeding was significantly higher in nonsmokers than in current smokers (1.6% and 1.1%,* P*=0.031; 7.2% and 6.1%,* P*=0.019). The rate of revascularization was significantly higher in current smokers than in nonsmokers (9.1% and 8.0%,* P*=0.037). Multivariable Cox regression indicated that, compared with nonsmokers, current smokers were not independently associated with all endpoints (all* P*>0.05).

**Conclusions:**

2-year all-cause death, MACCE, MI, revascularization, stroke, ST, and bleeding risk were similar between current smokers and nonsmokers in CAD patients undergoing PCI.

## 1. Introduction

Cigarette smoking is generally known as an important risk factor for pathogenesis of coronary artery disease (CAD), as well as prognosis [1–8]. Smoking cessation is recommended by international guidelines as one of crucial measurements for secondary prevention regardless of revascularization [9–12]. However, several studies have demonstrated a higher incidence of acute myocardial infarction (MI) but improved or neutral outcome after reperfusion among smokers than nonsmokers, which is termed the smoker's paradox [13–17]. Several small-sample cohort studies in Chinese CAD patients who underwent percutaneous coronary intervention (PCI) reported more nonfatal MI, but similar all-cause death risk in smokers than nonsmokers [18–20]. Therefore, it is uncertain whether the phenomenon of the smoker's paradox exists in Chinese population. Large data is limited up to now. This problem is of paramount importance for secondary prevention management for CAD patients. This study analyzed a large single-center sample in China to explain the impact of smoking state at baseline on long-term prognosis of CAD patients who received PCI.

## 2. Methods

### 2.1. Ethical Statement

Ethical approvals were obtained from the Fuwai Hospital Research Ethics Committees (No. 2013-449). The Institutional Review Board approved the study protocol and all patients signed written informed consent before the intervention, including full set of risk-informed consent and information use consent for scientific purposes.

### 2.2. Study Population

A total of 10724 consecutive cases with CAD who underwent PCI were enrolled from January to December 2013 in our center, the largest cardiovascular center of China. Smoking state at baseline was defined according to smoking history recorded at admission. Nonsmokers included patients who never smoked and those former smokers without cigarette usage in recent 3 months. The rest was defined as current smokers regardless of reduced quantity of smoking or quitting cigarette less than 3 months. Diagnosis of ST-segment elevated myocardial infarction (STEMI), non-ST-segment elevated myocardial infarction (NSTEMI), unstable angina pectoris (UAP), and stable coronary artery disease (SCAD) was in terms of criteria based on the “2013 European Society of Cardiology (ESC) guidelines on the management of SCAD,” “2015 ESC guidelines for the management of acute coronary syndromes (ACS) in patients presenting without persistent ST-segment elevation,” and “fourth universal definition of myocardial infarction (2018)” [9, 10, 21].

### 2.3. Procedural Details

Before selective PCI, if not taking long-term aspirin and clopidogrel, patients received 300 mg aspirin and P2Y12 inhibitor with loading dose orally. Patients with ACS scheduled for primary PCI received the same dose of aspirin and clopidogrel (loading dose 300 mg or 600 mg, according to bleeding risk) as soon as possible. Ticagrelor was seldom used in our center in the year of 2013, only when clopidogrel resistance was observed and patients were willing to take it on their own expense (loading dose of 180 mg or cumulative dose of 180 mg followed by 90 mg twice a day). Before coronary angiography (CAG), 3000 U heparin sodium was administered through an arterial sheath or intravenously. Before PCI, 100 U/kg of heparin sodium was administered. The dose was lowered to 50–70 U/kg in patients over the age of seventy to reduce bleeding risk. If PCI proceeded for more than 1 h, an additional 1000 U of heparin sodium was administered. Results of CAG were read by experienced cardiologists. More than 50% stenosis of left main artery (LM), left anterior descending artery (LAD), left circumflex artery (LCX), right coronary artery (RCA), and main branch of these vessels was defined as coronary artery stenosis. More than 70% stenosis of the vessels mentioned above, along with ischemic symptoms or ischemic evidence shown by examinations, was indicated for coronary stent implantation. Three-vessel disease (TVD) was defined as angiographic stenosis of ≥50% in all three main coronary arteries, LAD, LCX, and RCA. Synergy between Percutaneous Coronary Intervention with Taxus and Cardiac Surgery (SYNTAX) score (SS) and residual SNYTAX score (rSS) was assessed by two of the three experienced cardiologists in an independent angiographic core laboratory, who were blinded to clinical outcomes. High, intermediate, and low SS were defined as SS≥33, 23≤SS<33, and 0≤SS<23. Incomplete revascularization (ICR) was defined as rSS≥8, which was identified as a level strongly associated with increased cardiac death, MI, revascularization, and MACCE [22].

### 2.4. Follow-Up and Definitions

The patients were visited 30 days and 6 months after PCI and every 1 year thereafter. Information of in-hospital outcome was obtained through review of medical records, and the long-term clinical outcome was collected from survey completed by telephone follow-up, follow-up letter, or visit. A group of independent clinical physicians oversaw checking and confirmed all adverse events carefully. Investigators training, blinded questionnaire filling, and telephone recording were performed to control the data quality.

Primary endpoint was all-cause death. Composite endpoint was defined as major adverse coronary events (MACCEs), including all-cause death, revascularization, MI, and stroke. Secondary endpoints were MACCE, cardiac death, revascularization, MI, stroke, stent thrombosis (ST), and bleeding. Cardiac death is identified as death caused by MI, heart failure, and/or malignant arrhythmia definitely, or death which cannot be explained clearly by other reasons. ST was defined as definite, probable, and possible ST based on the Academic Research Consortium criteria. Bleeding was defined according to criteria established by Bleeding Academic Research Consortium (BARC), excluding BARC 0 and 1 types.

### 2.5. Statistical Analysis

Data statistics was applied using SPSS 22.0 (IBM Corp., Armonk, New York, USA). Student's t-tests were used to compare continuous variables while Chi-square tests were applied to compare categorical variables between the two groups. Kaplan–Meier curves were drawn to compare cumulative event rates of the two groups. Multivariate Cox proportional hazard regression analyses were applied to control baseline confounders. Covariates for Cox regression were those variables with significant differences in baseline or important clinical meaning. All P values were two sided with a significance level of 0.05. Tendency of significant difference was judged when 0.05<P<0.1.

## 3. Results

### 3.1. Baseline Characteristics

Among 10724 cases analyzed, current smokers and nonsmokers accounted for 57.1% and 42.9%, respectively. In current smokers, majority (96.0%) were male, which was significantly higher than that in nonsmokers (52.0%,* P<0.001*). The average age of current smokers was 4.2 years younger than that of nonsmokers. Current smokers had significantly higher BMI than nonsmokers (26.1 ± 3.2 and 25.8 ± 3.2,* P*<0.001). Current smokers had less comorbidities of hypertension (61.9% and 67.7%,* P*<0.001) and diabetes mellitus (DM) (29.2% and 31.6%,* P*=0.008), but more family history of CAD (26.4% and 22.5%,* P*<0.001), previous MI (22.1% and 15.3%,* P*<0.001), and prior PCI or CABG (28.1% and 23.7%,* P*<0.001) than nonsmokers. Current smokers had lower LVEF level (62.3 ± 7.4 and 63.3 ± 7.1,* P*<0.001), but higher eGFR (93.0 ± 14.7 and 89.0 ± 15.4,* P*<0.001) than nonsmokers. Angina pectoris happened less (70.6% and 78.5%,* P*<0.001), while STEMI occurred more, (16.2% and 10.0%,* P*<0.001) in current smokers than nonsmokers. Antiplatelet drugs and statin were similarly applied between 2 groups (*P*>0.05), while *β*-blocker and calcium antagonist were less prescribed at discharge in current smokers than nonsmokers (89.6% and 91.0%,* P*=0.018; 46.4% and 51.6%,* P*<0.001). (Table 1)

Current smokers were implanted with the second-generation drug-eluting stents (DES) less than nonsmokers (56.0% and 57.9%,* P*=0.043). Current smokers had lower SNYTAX score (11.5 ± 8.1 and 11.9 ± 8.1,* P*=0.019) and less LAD involved lesions (89.5% and 91.8%,* P*<0.001), but longer times of procedure than nonsmokers (37.4 ± 33.6 and 35.6 ± 28.5,* P*=0.002). (Table 1)

### 3.2. Clinical Outcomes and Subgroup Analysis

**Figure 1:**
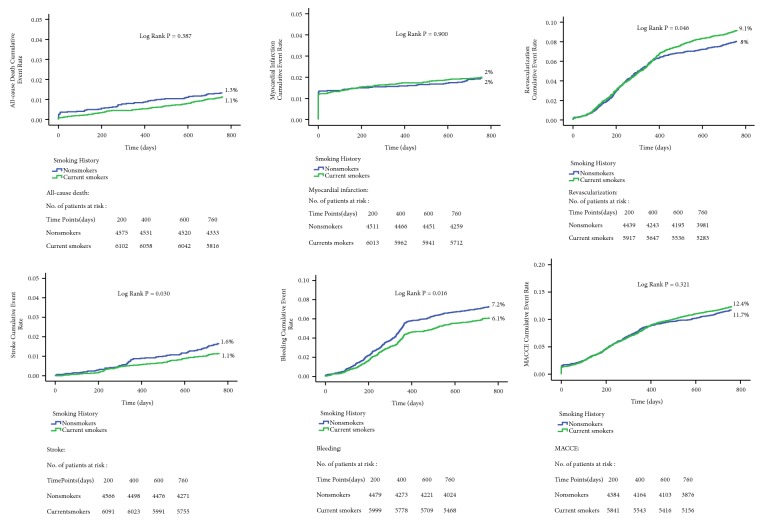
Kaplan-Meier survival curves between current smokers and nonsmokers.

Clinical follow-up was completed for 10665 patients (99.4%) of 2 years. The average follow-up was 872.4 days. The occurrence of adverse cardiovascular events in each group is listed in Table 2. During 2-year follow-up, the rates of all-cause death, MACCE, cardiac death, MI, and ST were not significantly different between the two groups (all* P*>0.05). But the rate of stroke and bleeding was significantly higher in nonsmokers than in current smokers (1.6% and 1.1%,* P*=0.031; 7.2% and 6.1%,* P*=0.019). The rate of revascularization was significantly higher in current smokers than in nonsmokers (9.1% and 8.0%;* P*=0.037). Kaplan-Meier curves revealed the same finding. (Figure 1)

Multivariable Cox regression analysis indicated that current smokers, compared with nonsmokers, were not independently associated with 2-year all-cause death (HR 1.03, 95%CI 0.69-1.54,* P* = 0.881), MACCE (HR 0.98, 95%CI 0.86-1.12,* P* = 0.766), and other all endpoints (all* P*>0.05). (Table 3)

**Figure 2:**
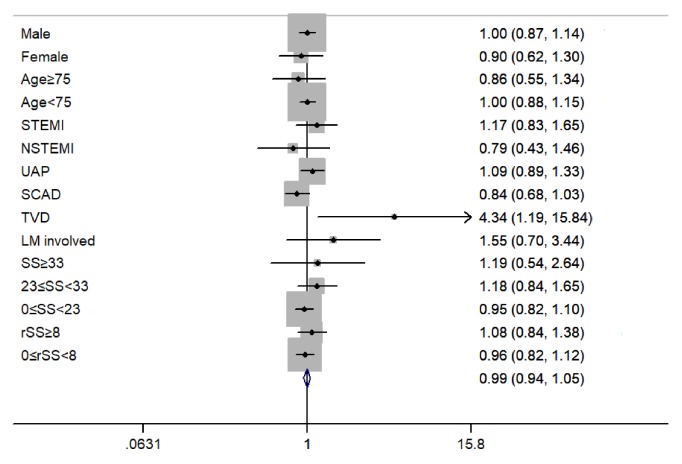
Forest plot of subgroup analysis. The multivariable analysis indicated whether current smoking is associated with 2-year MACCE.

Subgroups included male or female subgroups, patients with age≥75 or <75, patients diagnosed with STEMI, NSTEMI, UAP, or SCAD, patients with TVD or LM involved, patients with high, intermediate, or low SS, and patients who underwent CR or ICR. The interaction analysis showed classification of CAD was not independent of baseline smoking status (*P*=0.038). COX regressions analysis showed that, in all subgroups except for TVD subgroup, current smokers, compared with nonsmokers, were also not independently associated with MACCE (all* P*>0.05). In patients with TVD, current smokers were associated with significantly higher MACCE risk compared with nonsmokers (HR 4.34, 95%CI 1.19-15.85,* P*=0.026). (Figure 2)

## 4. Discussion

Due, in part, to the prothrombotic effects of smoking, cigarette smokers are more likely to present with STEMI. The “smoker's paradox” is discussed mainly in STEMI patients. The possible explanation is that smoking is associated with platelet aggregation and blood coagulability. Thus, coronary obstruction in smokers may be more thrombogenic and less atherosclerotic than in nonsmokers, leading more likely to be perfused spontaneously or by thrombolytic therapy [13–17, 23–26].

In this study, we have shown in a large contemporary cohort of patients with CAD undergoing PCI that (1) 2-year outcomes were similar between current smokers and nonsmokers; (2) MACCE risk was similar between current smokers and nonsmokers in all subgroups except for TVD subgroup. This finding indicated that the “smoker's paradox” also exists in CAD patients undergoing PCI in Chinese population. It is difficult to interpret that the results of impact of smoking on 2-year outcomes were not statistically significant. Possible explanation may focus on the damage mechanisms of cigarettes. The hypercoagulability of cigarette smokers may not only predispose them to the early occurrence of MI, but also could predispose them to reinfarction [15]. It was indeed observed in this study that smokers were associated with more STEMI and less angina pectoris than nonsmokers, and smoking status had significant interaction effect with classification of CAD. The hypercoagulability may be early inhibited by effective antithrombotic therapy. As the baseline medication analysis showed, antiplatelet drugs were highly prescribed in the two groups, showing no significant differences. Other pathological mechanisms shown in previous studies include an unfavorable modulation of autonomic cardiac control, leading to a shift towards sympathetic predominance accompanied by increased levels of catecholamines, lower arrhythmogenic threshold, increased vasoconstriction, and increased myocardial oxygen consumption [26–30]. The corresponding drug solutions involve appropriate *β*-blockers and calcium antagonists. The hazard of smoking may be offset by the comprehensive medication to some extent. Therefore, we think that this “neutral” result is inadequate to question the necessity of smoking cessation for secondary prevention. Cardiologists should continue to emphasize the pivotal role of smoking cessation in risk reduction especially in patients with established CAD.

On the other hand, both supporters and opponents of “smoker's paradox” found that smokers were associated with younger age, more male sex, and less comorbidities compared with nonsmokers. Some researchers indicated that the phenomenon of “smoker's paradox” could be partly explained by fewer coexisting high-risk features in patients with AMI who are currently smoking [13–17, 23–25, 31, 32]. In our study, the “lower risk” of stroke and bleeding in current smokers may be partly explained in a similar fashion to that seen in patients with AMI, that baseline characteristics do play important role in data interpretation. Current smokers were associated with younger age, predominant of male sex, less DM, and hypertension. All these factors may reduce stroke and bleeding risk and be considered as confounders.

The results also showed that current smokers were associated with more revascularization than nonsmokers in univariate analysis, but the correlation was no longer significant after multivariate adjustment. It means that the explanation lied in baseline differences. However, except for less second-generation durable polymer DES implantation in current smokers, baseline analysis showed better general conditions and less severe lesions of current smokers than nonsmokers, revealed by more male, younger age, less DM and hypertension, higher eGFR, lower SNYTAX scores, and less LAD involved lesions, which cannot answer why current smokers had increased revascularization rate. It is worth noting that current smokers had higher BMI, higher urine acid level, and more family history of CAD than nonsmokers. It is indicated that possibly genetic and metabolic mechanisms are involved in the relationship between smoking and revascularization after PCI. As known, smokers are always a group of people keeping ill habits. Genetic susceptibility and unhealthy way of life may explain current smokers' increased revascularization rate to some extent. Interestingly, current smokers also had more previous MI and prior PCI or CABG history. The repeated hospitalization and revascularization did not convert to mortality; however, they inevitably contributed to increased burden of governmental health fund.

Additionally, patients with LM involved or TVD were not different from current smokers and nonsmokers in this study, not consistent with previous data. However, in subgroup of patients with TVD, current smokers had about 4.3 times MACCE risk than nonsmokers. Because event rates of all-cause death, MI, and stroke were low in TVD subgroup, COX regression models were not significant. We could not find out which endpoint current smoking really effects. As reported in the Chinese postmenopausal women, current smoking and the presence of multiple-vessel disease can independently predict events of all-cause death, nonfatal infarction, or unstable angina [33]. It is reasonable to speculate that smoking has effect on outcomes of patients who suffered from severe CAD.

In general, this study analyzed the association between baseline smoking status and 2-year outcomes in a large-sample cohort with CAD who underwent PCI, expecting to find some adverse impacts of smoking as routing perception. Unexpectedly, the results were not statistically significant. How to interpret the results for clinical practice. Maybe we should be conscious of the complicated clinical situation that smoking possibly plays more important role in initial triggering mechanisms in ACS, due to vasoconstriction, hypercoagulability, platelet aggregation, and endothelial dysfunction [13–15, 23–30]. However, the progression of atherosclerosis can be influenced by multifactors, including genetic mechanisms and secondary medication [34, 35]. Genetic factors were not included in most clinical studies in this area for uncertainty up to date. And, secondary medication may be of conversing effect of smoking to some extent. Antiplatelet and anticoagulation therapy may reverse the platelet aggregation and hypercoagulability driven by tobacco. *β*-blockers and calcium antagonists may decrease oxygen consumption and relieve vasoconstriction. And statins may protect endothelium and anti-inflammation. The considerate secondary medication for patients who receive PCI in our center may explain the results in this study to some extent. Further research is required to demonstrate the speculation.

Several limitations should be taken into consideration. Firstly, former smokers were much less than current smokers and never smokers, which made comparisons among current smokers, former smokers, and never smokers lack statistical power. Therefore, former smokers and never smokers were included into one group. The heterogeneity between former smokers and never smokers may affect results to some extent. Secondly, no data was available regarding the quantity or duration of smoking. Thus, we cannot analyze dose-response or duration-response relationship between smoking and adverse events risk. Thirdly, no data was available on those who quit smoking after PCI, and how their event rate is compared to that of patients who continued to smoke. Finally, there may be additional confounders that are not controlled for within our model. Nevertheless, this is a large core laboratory analysis comparing current smokers and nonsmokers in patients with whole CAD spectrum in Chinese population, in terms of both outcomes and angiographic data, and we believe that we have accounted for the most clinically relevant variables in our model.

## 5. Conclusions

2-year all-cause death, MACCE, MI, revascularization, stroke, ST, and bleeding risk were all similar between current smokers and nonsmokers in CAD patients undergoing PCI. Subgroup analysis found that current smokers were associated with higher MACCE risk in patients with TVD.

## Figures and Tables

**Table 1:** The baseline clinical, angiographic, and procedural characteristics and medication situation.

Variables	All (n = 10724)	Current smokers (n = 6123)	Nonsmokers (n = 4601)	P value
*Demographic characteristics*				
Male gender, %	8272 (77.1)	5880 (96.0)	2392 (52.0)	< 0.001
Age, years	58.4 ± 10.3	56.6 ± 10.0	60.8 ± 10.2	< 0.001
BMI, kg/m2	25.9 ± 3.2	26.1 ± 3.2	25.8 ± 3.2	< 0.001
*Coexisting conditions, *%				
Hypertension	6906 (64.4)	3789 (61.9)	3117 (67.7)	< 0.001
DM	3238 (30.2)	1786 (29.2)	1452 (31.6)	0.008
Hyperlipidemia	7211 (67.2)	4138 (67.6)	3073 (66.8)	0.387
Previous MI	2061 (19.2)	1355 (22.1)	706 (15.3)	< 0.001
Prior PCI or CABG	2808 (26.2)	1719 (28.1)	1089 (23.7)	< 0.001
Family history of CAD	2651 (24.7)	1614 (26.4)	1037 (22.5)	< 0.001
CVD	1150 (10.7)	643 (10.5)	507 (11.0)	0.391
PVD	288 (2.7)	177 (2.9)	111 (2.4)	0.129
COPD	247 (2.3)	143 (2.3)	104 (2.3)	0.799
LVEF (%)	62.8 ± 7.4	62.3 ± 7.4	63.3 ± 7.1	< 0.001
*Clinical presentation, *%				
Asymptomatic ischemia	869 (8.1)	523 (8.5)	346 (7.5)	< 0.001
Stable angina	3424 (31.9)	1842 (30.1)	1582 (34.4)	< 0.001
Unstable angina pectoris	4509 (42.0)	2479 (40.5)	2030 (44.1)	< 0.001
AMI	1922 (17.9)	1279 (20.9)	643 (14.0)	< 0.001
STEMI	1447 (13.5)	989 (16.2)	458 (10.0)	< 0.001
NSTEMI	475 (4.4)	290 (4.7)	185 (4.0)	0.075
*Laboratory examination*				
eGFR before PCI, mL/min/1.73m2	91.3 ± 15.1	93.0 ± 14.7	89.0 ± 15.4	< 0.001
HGB before PCI, g/L	141.0 ± 15.8	144.8 ± 14.4	136.1 ± 16.2	< 0.001
PLT before PCI, 10^9^/L	203.6 ± 54.4	201.4 ± 53.6	206.4 ± 55.4	< 0.001
Urine acid, *μ*mol/L	341.6 ± 84.7	353.2 ± 82.1	326.2 ± 85.7	< 0.001
HbA1c, %	6.6 ± 1.2	6.6 ± 1.2	6.6 ± 1.3	0.029
LDL-C, mmol/L	2.50 ± 0.90	2.48 ± 0.88	2.54 ± 0.93	< 0.001
ESR, mm/h	10.8 ± 11.3	9.3 ± 10.2	12.9 ± 12.3	< 0.001
*Angiographic and procedural characteristics*				
SNYTAX score	11.7 ± 8.1	11.5 ± 8.1	11.9 ± 8.1	0.019
Residual SNYTAX	3.4 ± 5.7	3.4 ± 5.6	3.5 ± 5.8	0.334
LM or TVD, %	457 (4.3)	267 (4.4)	190 (4.1)	0.558
LAD involved, %	9702 (90.5%)	5480 (89.5)	4222 (91.8)	< 0.001
No. of target lesions	1.40 ± 0.66	1.41 ± 0.67	1.39 ± 0.65	0.098
No. of stents per patient	1.80 ± 1.11	1.82 ± 1.13	1.79 ± 1.08	0.197
Time of procedure, min	36.7 ± 31.5	37.4 ± 33.6	35.6 ± 28.5	0.002
*Procedure and stent type, *%				0.019
PTCA	237 (2.2)	121 (2.0)	116 (2.5)	
BMS	64 (0.6)	30 (0.5)	34 (0.7)	
First-generation durable polymer DES	597 (5.6)	363 (5.9)	234 (5.1)	
Second-generation durable polymer DES	6094 (56.8)	3428 (56.0)	2666 (57.9)	
Domestic biodegradable polymer DES	1572 (14.7)	925 (15.1)	647 (14.1)	
Mixed implantation of DES	1692 (15.8)	976 (15.9)	716 (15.6)	
Others (Janus, Yinyi)	167 (1.6)	93 (1.5)	74 (1.6)	
Procedure unsuccess	301 (2.8)	187 (3.1)	114 (2.5)	
*Medication at discharge, *%				
Aspirin	10585 (98.7)	6047 (98.8)	4538 (98.6)	0.562
Clopidogrel	10701 (99.8)	6114 (99.9)	4587 (99.7)	0.081
Ticagrelor	19 (0.2)	9 (0.1)	10 (0.2)	0.391
DAPT	10583 (98.7)	6047 (98.8)	4536 (98.6)	0.440
Statin	10285 (95.9)	5878 (96.0)	4407 (95.8)	0.578
Calcium antagonist	5216 (48.6)	2842 (46.4)	2374 (51.6)	< 0.001
*β*-blocker	9673 (90.2)	5487 (89.6)	4186 (91.0)	0.018

AMI: acute myocardial infarction; BMI: body mass index; BMS: bare metal stent; CAD: coronary artery disease; CABG: coronary artery bypass grafting; COPD: chronic obstructive pulmonary disease; CVD: cerebral vascular disease; DES: drug-eluting stent; DM: diabetes mellitus; DAPT: dual antiplatelet therapy; eGFR: estimated glomerular filtration rate; ESR: erythrocyte sedimentation rate; HGB: hemoglobin; HbA1c: hemoglobin A1c; LAD: left anterior descending artery; LM: left main; LDL-C: low density lipoprotein cholesterol; LVEF: left ventricular ejection fraction; MI: myocardial infarction; NSTEMI: non-ST-segment elevated myocardial infarction; PCI: percutaneous coronary intervention; PLT: platelet; PTCA: percutaneous transluminal coronary angioplasty; PVD: peripheral vascular disease; STEMI: ST-segment elevated myocardial infarction; SYNTAX: Synergy between Percutaneous Coronary Intervention with Taxus and Cardiac Surgery; TC: total cholesterol; TVD: three-vessel disease.

Data are expressed as mean ± standard deviation, or counts (percentage).

**Table 2:** 2-year outcomes.

Endpoints	All (n = 10724)	Current smokers (n = 6123)	Nonsmokers (n = 4601)	P value
All-cause death	131 (1.2)	70 (1.1)	61 (1.3)	0.394
MACCE	1295 (12.1)	757 (12.4)	538 (11.7)	0.292
Cardiac death	74 (0.7)	39 (0.6)	35 (0.8)	0.444
Myocardial infarction	212 (2.0)	122 (2.0)	90 (2.0)	0.893
Revascularization	923 (8.6)	557 (9.1)	366 (8.0)	0.037
Stent thrombosis	91 (0.8)	53 (0.9)	38 (0.8)	0.825
Stroke	145 (1.4)	70 (1.1)	75 (1.6)	0.031
Bleeding	702 (6.5)	371 (6.1)	331 (7.2)	0.019

**Table 3:** Multivariate Cox regression analysis in whole cohort, SCAD subgroup, and ACS subgroup.

Endpoints	Whole cohort		SCAD subgroup		ACS subgroup	
	HR (95%CI)	P value	HR (95%CI)	P value	HR (95%CI)	P value
All-cause death	1.03 (0.69, 1.54)	0.881	0.81 (0.41, 1.60)	0.537	1.12 (0.68, 1.85)	0.662
MACCE	0.98 (0.86, 1.12)	0.766	0.84 (0.68, 1.03)	0.089	1.09 (0.93, 1.29)	0.298
Cardiac death	1.16 (0.68,1.98)	0.587	0.42 (0.14, 1.30)	0.133	1.72 (0.91, 3.28)	0.098
Myocardial infarction	0.90 (0.65, 1.25)	0.547	0.77 (0.46, 1.29)	0.321	1.00 (0.65, 1.52)	0.98
Revascularization	0.97 (0.83, 1.13)	0.687	0.81 (0.64, 1.04)	0.097	1.10 (0.90, 1.34)	0.362
Stent thrombosis	1.03 (0.64, 1.65)	0.92	0.52 (0.22, 1.25)	0.143	1.47 (0.82, 2.64)	0.196
Stroke	1.17 (0.79, 1.71)	0.434	1.07 (0.57, 2.03)	0.83	1.26 (0.78, 2.03)	0.349
Bleeding	0.90 (0.75, 1.08)	0.273	0.96 (0.73, 1.27)	0.763	0.85 (0.66, 1.08)	0.183

ACS: acute coronary syndrome; SCAD: stable coronary artery disease.

## Data Availability

The data used to support the findings of this study are available from the corresponding author upon request.
